# COVID-19 pneumonia in a child with hepatic encephalopathy: A case study

**DOI:** 10.22037/ijcn.v15i1.30879

**Published:** 2021

**Authors:** Naghi DARA, Naghmeh SHARIFI, Masoud GHANBARI BOROUJENI, Amirhossein HOSSEINI, Aliakbar SAYYARI

**Affiliations:** 1 Pediatric Gastroenterology, Hepatology, and Nutrition Research Center, Research Institute for Children’s Health, Shahid Beheshti University of Medical Sciences, Tehran, Iran; 2 Resident of pediatrics, Shahid Beheshti University of Medical Sciences, Tehran, Iran.; 3 Medical Student, Shahid Beheshti University of Medical Sciences, Tehran, Iran.

**Keywords:** COVID-19, children, pneumonia, chronic liver cirrhosis, hepatic encephalopathy

## Abstract

Coronavirus disease 2019 (COVID-19) is caused by the seventh coronavirus, known as the severe acute respiratory syndrome coronavirus 2(SARS-CoV-2). Children often have milder diseases than adults with very rare mortality. Gastrointestinal manifestations and a mild increase in liver enzymes have been reported in 8.8% to 53% of COVID-19 cases. However, liver failure is extremely rare and has not been reported so far in the literature. The prevalence of comorbidities is not clear in children with COVID-19. Here, we reported a fatal case of simultaneous pneumonia secondary to SARS-CoV-2and acute liver failure in a 14-year-old boy with liver cirrhosis.

## Introduction

The severe acute respiratory syndrome coronavirus 2 (SARS-CoV-2) was first identified in Wuhan, China, in December 2019 and then spread rapidly around the world. It became the causative agent of a respiratory syndrome named coronavirus disease 2019 (COVID-19) (1). SARS-CoV-2 mostly involves the respiratory system, although it affects other organs, including the gastrointestinal tract, liver, kidney, and skin (2). As reported, the severity of COVID-19 is directly correlated with the patient’s age and several comorbidities. However, no data are currently available regarding the clinical outcome in pediatric patients with chronic liver diseases (3). The mortality rate of COVID-19 has been reported around 3% in the general population, and most COVID-19 patients have been cured quickly (3).Children often have milder diseases than adults with extremely rare mortality(4). Despite the fact that COVID-19 diagnosis depends on virus genome RNA RT-PCR, a chest CT scan is useful in the early detection of pulmonary involvements before the onset of symptoms (5). Several studies reported gastrointestinal symptoms such as nausea, vomiting, diarrhea, and liver function abnormalities during the course of COVID-19 (6). This is a very rare presentation of liver involvement in COVID-19. The interaction between COVID-19 and liver function is still unclear, and more investigations should be carried out to clarify this interaction. Here, we presented a fatal case of superimposed COVID-19 pneumonia on chronic liver cirrhosis with a dominant feature of encephalopathy and acute liver failure.

In this report, we realized that a pediatric gastroenterologist and a pediatric neurologist need to consider liver involvement as a primary presentation of COVID-19. Moreover, a physician should consider acute liver failure in a patient with chronic liver disease as a complication of this viral infection.

## Case presentation

A 14-year-old boy, G2P2L2A0, presented with an abdominal protrusion, lower extremities pitting edema, icter, and weight gain. He was under observation of pediatric nephrologists with an impression of nephrotic syndrome due to proteinuria for40 days. Due to abnormal liver function tests with prolonged prothrombin time and INR, he was transferred to our hospital. Three days before admitting to our hospital, he developed a dry cough, ascites, and an increase in yellowish discoloration of skin and sclera. Before admission, he had a weight gain of about 8 kg. He had no history of fever, nausea, vomiting, oligouria, myalgia, and dyspnea, although he was in contact with his family members with cough.

At the time of admission, he was alert with GCS=15, and his sclera and skin were icteric. The patient was ill and toxic looking. Physical examination of the patients revealed a body temperature of36.7^°^C, a respiratory rate of 18/min, a blood pressure of 110/70 mmHg, and a heart rate of 84 beats/min. Fine rales were heard in the left lung. The spleen was palpable 6cm below the costal margin and, the liver was detected about 8cm below the costal margin with a firm consistency. The abdomen was protruded due to moderate ascites. Stigmata of chronic liver disease such as spider hemangioma, palmar erythema, and caput medusa were observed in the abdominal examination. The lower extremities had 4+ pitting edema. Laboratory investigations revealed abnormal coagulation profile, direct hyperbilirubinemia, liver enzyme elevation, mild anemia with thrombocytopenia, hypophosphatemia, hyponatremia, hypomagnesemia, and hypokalemia. Laboratory findings are summarized in Table1. Serologic markers for viral hepatitis A, B, and C viruses, HIV, and cytomegalovirus were negative. The autoimmune hepatitis profile, ceruloplasmin, and 24-hr urine copper results are summarized in Table2. There was no specific key point in his past medical or family history.

**Figure 1:**
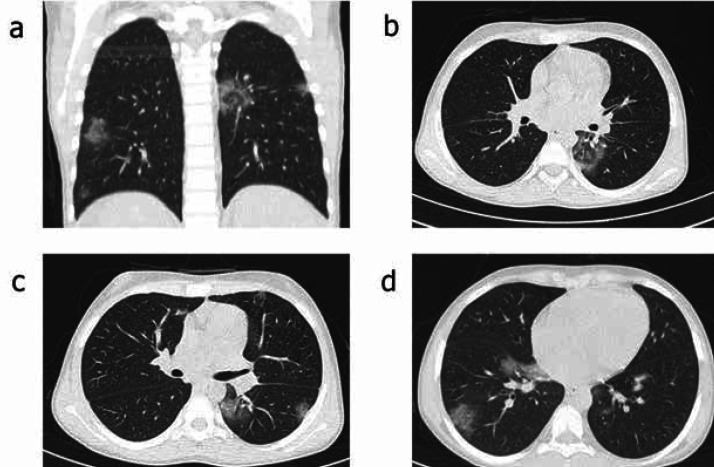
The chest CT scan without contrast in a 14-year-old boy with chronic liver cirrhosis. There are bilateral multifocal sub-pleural ground-glass opacities with mild pleural effusion highly suggestive for COVID-19

Chest radiography revealed diffuse haziness through the left lung field. A spiral chest CT scan without contrast showed bilateral multifocal sub-pleural ground-glass opacity with mild pleural effusion highly suggestive for COVID-19 (Figure 1). Abdominopelvic ultrasonography revealed hepatomegaly with coarse echogenicity and moderate to severe ascites in favor of liver cirrhosis. The first complete blood count (CBC) showed anemia, lymphopenia, and thrombocytopenia. Table 3summarizesserial CBC. The nasopharyngeal swab sample RT-PCR confirmed the diagnosis of COVID-19 pneumonia. The serial nasopharyngeal sample test for COVID-19 was positive for three consecutive weeks. The patient was treated with non-invasive ventilation, hydroxychloroquine (200mg BID), and supportive drugs, including ursodeoxycholic acid 250mg TDS, vitamin K intravenous 10mg daily, vitamin E 200 mg oral daily, spironolactone 25mg BD orally, albumin, calcium gluconate, glycophose, intravenous cefotaxime, cryoprecipitate, fresh frozen plasma, and fresh packed-cell. The results of several abdominal paracenteses were transudative and clear. The patient had an absolute indication for emergency liver transplantation, and despite an initial improvement, his condition subsequently worsened, and he gradually developed encephalopathy with mood and mental status changes such as day time sleeping, lethargy, tremor, and respiratory distress. Unfortunately, the patient died after 18 days of hospitalization with features of refractory pulmonary hemorrhage.

## Discussion

Several studies documented a mild increase in serum aminotransferase (14-53%) in many COVID-19 patients (6). This condition is similar to the past SARS outbreak, with liver impairments in up to 60% of infected patients (7). Furthermore, the prevalence of chronic liver disease is about 2-11%in COVID-19 patients. Previous studies showed that pre-existing liver disease increased COVID-19 mortality as well as other chronic diseases like hypertension, cardiovascular disease, and diabetes mellitus (6). Evaluations showed that co-infection with COVID-19 and hepatitis B virus resulted in adverse outcomes and higher mortality (8).Hepatic dysfunction in COVID-19 can be the result of direct liver infection, as the virus is extracted from stool samples. Immune-mediated inflammation and uncontrolled cytokine production, drug toxicity, and hypoxia are other probable mechanisms of liver injury in COVID-19 patients (9).

SARS-CoV-2affects the target organ by binding to the angiotensin-converting enzyme 2 receptor, which has been suggested to be expressed in liver and bile duct cells (6). Thus, SARS-CoV-2 may directly damage liver cells and cause clinical and laboratory signs of liver failure. Some studies mentioned the increased serum level of lactate dehydrogenase (LDH), aspartate aminotransferase (AST), alanine aminotransferase (ALT), gamma-glutamyl transferase (GGT), bilirubin, and alkaline phosphatase (ALP) in COVID-19 patients, and a greater level of ALT was related to worse prognosis (8, 10).

Our case had idiopathic chronic liver cirrhosis and was a candidate for liver transplantation. In the outbreak of COVID-19, the patient’s clinical condition deteriorated, and he developed ascites, icter, and increased liver enzymes. Gradually, a chest CT scan showed dyspnea and encephalopathy symptoms with pulmonary abnormalities in the patient. Finally, he expired despite receiving treatment.

In conclusion, based on the evidence previously presented, liver failure has not been reported in COVID-19 patients, and it is only a presentation of multisystem inflammatory syndrome in children (MISC). Thus, unexplained liver dysfunction should be considered as a probable sign of complicated COVID-19 or MISC. To understand this, a screening test needs to be performed as soon as possible. It can be helpful in the early diagnosis and treatment of COVID-19 patients and also in the prevention of further morbidity and mortality.

**Table1:** Primary Laboratory findings*

**Marker**		Value on admission	Value in follow up	Marker	Value on admission	Value in follow up	Value in follow up
**Na(mEq/L)**	133		131	TP (g/dL)	5.5	6.1	6
**K (mEq/L)**	3.5		3.1	Alb (g/dL)	2.1	3.3	3.1
**Ca(mg/dL)**	7.2		7.6	AST(IU/dL)	392	58	100
**P (mg/dL)**	2		2.1	ALT(IU/dL)	234	92	108
**Mg(mg/dL)**	1.5		1.3	TB (mg/dL)	8.7	40	41
**BS(mg/dL)**	68		57	DB (mg/dL)	3.5	19.9	21
**BUN(mg/dL)**	7		8.5	Alkp (IU/L)	119	253	266
**Cr (mg/dL)**	0.2		0.4	LDH(mg/dL)	813	-	-
**Ferritin(ng/dL)**	579		-				

*Abbreviations: ALT, alanine transaminase; AST, aspartate aminotransferase; TP, total protein; Alb, albumin; TB, total bilirubin; DB, direct bilirubin; Na, sodium; K, potassium; BS, blood sugar; BUN, blood urea nitrogen; Ca, calcium; Cr, creatinine; Mg, magnesium; P, phosphate; LDH, lactate dehydrogenase.

**Table 2:** Advanced Specific Tests for Liver Disease *

Marker	Value	Marker	Value	Marker	Value
**ANA**	1/20	Ceruloplasmin (mg/dL)	30	HAV- Ab (IgM)	0.4
**AMA**	<1/10	24hr Urine copper (micg/dL)	25	HBS-Ag (ECL)	0.8
**ASMA**	<1/10	IgG (g/L)	16	HBs-Ab (ECL)	<5
**ALKM1**	<1/10	HCV-Ab (ECL)	0.5	HIV -Ab (ECL)	0.6

*Abbreviation: ANA, anti-nuclear antibody; AMA, anti-mitochondrial antibody; ASMA, anti-smooth muscle antibody; Anti-LKM1, anti-liver kidney microsome type 1 antibody; ECL, electrochemiluminescence; HAV Ab, hepatitis A virus antibody; HBs Ab, hepatitis B surface antibody; HBs Ag, hepatitis B surface antigen; HCV-Ab, hepatitis C virus antibody; and IgM, immunoglobulin M; IgG, immunoglobulin G; HIV, human immune deficient virus.

**Table 3:** Complete Blood Count and Coagulation Profile*

**Marker**	Value 1	Value 2	Value 3	Value 4
WBC(*10^6^/L)	6300	3000	3400	14300
Hb (g/dL)	10.5	6.3	8	7.2
MCV (fL)	112	115	110	113
Plt(*10^6^/L)	78000	46000	53000	69000
PMN (%)	76%	67	74	91
Lymph (%)	19%	28	18	7
PT (S)	37	30.7	24	28
INR	5.6	4.2	2.9	3.2
PTT (S)	50	75	66	80

*Abbreviations: Hb, hemoglobin; WBC; white blood cell; INR, international normalized ration; MCV, mean cell volume; PT, prothrombin time; PTT, partial thromboplastin time; Plt, platelet; PMN, polymorphonuclear; Lymph, lymphocyte.
